# The Dual Role of Mesenchymal Stromal Cells and Their Extracellular Vesicles in Carcinogenesis

**DOI:** 10.3390/biology11060813

**Published:** 2022-05-25

**Authors:** Zarema Gilazieva, Aleksei Ponomarev, Albert Rizvanov, Valeriya Solovyeva

**Affiliations:** Institute of Fundamental Medicine and Biology, Kazan Federal University, 420008 Kazan, Russia; zaregilazieva@kpfu.ru (Z.G.); alesponomarev@stud.kpfu.ru (A.P.); albert.rizvanov@kpfu.ru (A.R.)

**Keywords:** mesenchymal stromal cells, extracellular vesicle, tumor microenvironment, intercellular communication, carcinogenesis, immunosuppression, neoangiogenesis, epithelial–mesenchymal transition, apoptosis, metastasis

## Abstract

**Simple Summary:**

Extracellular vesicles (EVs) are membrane structures that play the role of intermediaries between tumor cells and the tumor microenvironment (TME) because they have the ability to transport lipids, transcription factors, mRNA, and proteins. Mesenchymal stem cells (MSCs) are a major component of the TME and may have different effects on tumor progression using EVs. This review includes information about various studies which have reported that EVs from MSCs can have either antitumor or pro-tumor effects, depending on both the tumor type and developmental stage. It provides an overview of the published data on EV MSCs and their effect on tumor cells. In addition, the use of EV MSCs for the development of new methods for treating oncological diseases is described.

**Abstract:**

Mesenchymal stem cells (MSCs) are a major component of the tumor microenvironment (TME) and play an important role in tumor progression. MSCs remodel the extracellular matrix, participate in the epithelial–mesenchymal transition, promote the spread of metastases, and inhibit antitumor immune responses in the TME; however, there are also data pertaining to the antitumor effects of MSCs. MSCs activate the cell death mechanism by modulating the expression of proteins involved in the regulation of the cell cycle, angiogenesis receptors, and proapoptotic proteins. One of the main ways in which MSCs and TME interact is through the production of extracellular vesicles (EVs) by cells. Currently, data on the effects of both MSCs and their EVs on tumor cells are rather contradictory. Various studies have reported that EVs from MSCs can have either antitumor or pro-tumor effects, depending on both the tumor type and developmental stage. In this review, we discuss published data on EV MSCs and their effect on tumor cells. The molecular composition of vesicles obtained from MSCs is also presented in the review. In addition, the use of EV MSCs for the development of new methods for treating oncological diseases is described.

## 1. Introduction

Mesenchymal stem cells (MSCs) are a population of cells capable of self-renewal and differentiation into osteoblasts, chondrocytes, adipocytes, myocytes, and neurons [1,2]. MSCs can be obtained from various tissues, including bone marrow, adipose tissue, dental pulp, the placenta and the umbilical cord [3]. MSCs have been actively studied and used as a therapeutic tool for a long time, for many diseases, because they are able to undergo self-renewal, multipotency, and immunomodulation, in the absence of major histocompatibility complex (MHC) class II antigens [4,5]. Undoubtedly, MSCs play a special role in tumor progression; however, the mechanisms of action of MSCs on tumor cells, which include both anti-tumor and pro-tumor effects, are still being investigated.

MSCs are able to exhibit a pronounced tropism for areas of inflammation and tumor niches, where they become an integral part of the tumor stroma [6]. To demonstrate the tropism of MSCs for tumor niches, Nakamizo et al. used a mouse xenograft model of human glioma where MSCs, labeled with a fluorescent vital dye SP-DiI, were injected into the carotid artery. The cells were not found in healthy brain tissue, but were localized within areas of glioma, whereas fibroblasts did not show such specificity, and this indicated the MSCs were able to undertake selective migration to the area of tumor formation [7]. In addition, magnetic resonance imaging (MRI) was used to show MSC tropism in a rat orthotopic xenograft model of malignant glioma [8]. This tropism may be related to the monocyte chemoattractant protein 1 (MCP-1)/CC motif chemokine ligand 2 (CCL2) and stromal cell-derived factor-1 (SDF-1)/CXC motif chemokine ligand 12 (CXCL12) chemokines, which mediate MSC migration to CD133+ glioblastoma cells in vitro [9]. MSC tropism can be monitored using bioluminescence imaging, in which MSCs are modified with a luciferase gene. This method allows researchers to determine the migration and engraftment of MSCs in the area of inflammation or tumor formation [10].

The fact that MSCs have the capacity to migrate to tumor niches has been confirmed by a number of other studies. For example, human MSCs genetically modified with an oncolytic virus migrated to tumor cells and released viral particles, which then infected U87MG glioma cells in vitro. By using MSCs, it was possible to deliver 46 times more viral copies to the tumor area when compared with injecting a pure oncolytic virus [11]. The tropism of MSCs for tumor foci was also shown during systemic administration into xenograft mouse models with human mammary and thyroid gland tumors [12]. It is likely that MSCs, similar to immune cells, are able to migrate to inflammation foci due to chemotaxis [13]. Chemoattractants are mediators secreted by tumor cells, such as vascular endothelial growth factor (VEGF) and transforming growth factor (TGF) β1, as well as the cytokines interleukin (IL)-8 and neurotrophin-3 (NT-3) [14,15,16], which attract MSCs to the tumor microenvironment (TME).

**Figure 1:**
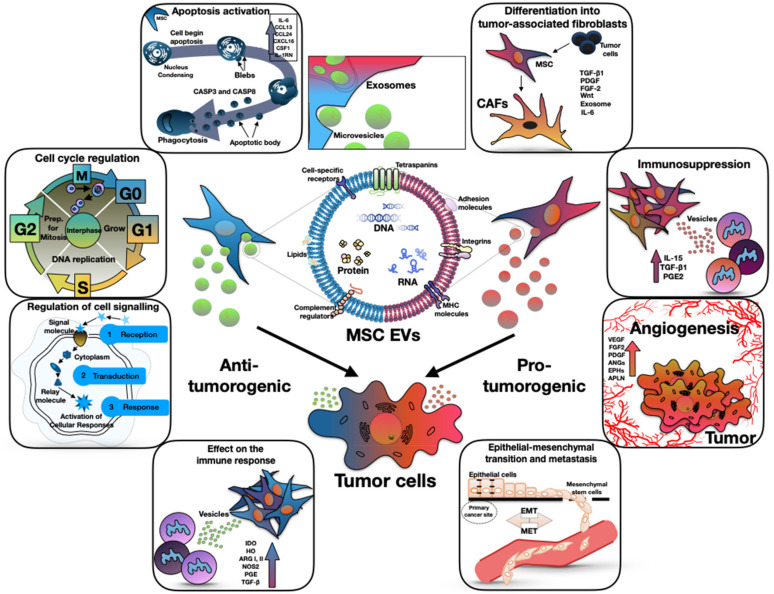
The dual (anti-tumorigenic and pro-tumorigenic) effect of mesenchymal stem cell derived extracellular vesicles on tumor progression. The central part of the figure shows the structure of the EVs. The left side of the figure shows the antitumor effect of EVs through the apoptosis activation, cell cycle regulation, effect on the immune response, and regulation of cell signaling. The right side of the figure shows the pro-tumor effect of EVs through the differentiation into tumor-associated fibroblasts, immunosuppression, angiogenesis and epithelial–mesenchymal transition.

Tumor cells can change the functional profile of MSCs from normal trophic to pro-tumor, using extracellular vesicles (EVs). This change leads to the fact that EVs from reprogrammed MSCs begin to affect other TME cells, such as fibroblasts, immune and endothelial cells [17]; however, there is evidence that MSCs also have the potential to exhibit antitumor activity, which is also mediated by EVs (Figure 1) [17].

## 2. Pro-Tumor Properties of Mesenchymal Stromal Cells

### 2.1. Differentiation into Tumor-Associated Fibroblasts

Tumor-associated fibroblasts (TAFs) in TMEs play a key role in tumor progression. TAFs are cells with a mesenchymal origin that are present in many invasive solid tumors and secrete large numbers of EVs. MSCs in the TME are able to differentiate into TAFs under the influence of various trophic factors, such as TGF-β and epidermal growth factor (EGF), secreted by tumor cells in large quantities [18,19]. In addition, the CXC motif chemokine receptor (CXCR) type 6 signaling pathway can stimulate the differentiation of MSCs into TAFs [20], whereas TGF-β can induce fibroblast differentiation into myofibroblasts by activating the TGF-β/SMAD family member 3 (SMAD3) signaling pathway, synthesis of α-smooth muscle actin (α-SMA) and basic fibroblast growth factor (FGF2) [21]. In a study by Miyazaki et al., in which a model of pancreatic cancer was reproduced, the origin of TAFs from MSCs was confirmed. Mice underwent transplantation with human MSCs extracted from adipose tissue and human pancreatic duct adenocarcinoma cells. It was shown that TAFs are derived from transplanted MSCs and are composed of heterogeneous TAF subpopulations [22]. MSCs can also differentiate into separate TAF subtypes depending on exposure to differing co-culture conditions [23].

TAFs allow the formation of a unique microenvironment that is important during tumor development by releasing fibronectin (FN) and collagen [24]. TAFs are able to stimulate tumor cell proliferation through the secretion of various growth factors, hormones, and cytokines, including hepatocyte growth factor (HGF), FGFs, SDF-1, and IL-6 [25]. SDF-1 activates CXCR4 synthesis and stimulates tumor cell proliferation [24,26]. TAFs also secrete chemokines such as insulin-like growth factor (IGF) 1 and 2, platelet-derived growth factor (PDGF), integrin-α11 (ITGA11), transmembrane heparan sulfate proteoglycan (syndecan-1 (SDC-1), CD138), and matrix metallopeptidase 2 (MMP2). These components have been shown to stimulate proliferation, migration and invasion of tumor cells, as well as increase tumor cell viability against the background of antitumor therapy, thus contributing towards the formation of resistant clones [27]. In in vitro experiments, TAFs have also increased the proliferation rates of neuroblastoma and colon cancer tumor cells [28,29].

Borriello et al. isolated cells from a primary human neuroblastoma tumor and characterized a population of TAFs secreting fibroblast activation protein α (αFAP) and fibroblast-specific protein 1 (FSP-1), which had similar phenotypic and functional characteristics to MSCs extracted from bone marrow [28]. Human neuroblastoma tumor analysis also revealed αFAP- and FSP-1-positive cells in the tumor stroma, which correlated with the presence of tumor-associated macrophages (TAMs). Presumably, the pro-oncogenic role of these TAFs might be mediated by activation of the signal transducer and activator of transcription 3 (STAT3) and extracellular signal-regulated protein kinase (ERK) signaling pathways 1/2 [28].

Various signaling pathways can be activated by tumor cell exosomes that act on MSCs. It has been shown that lymphocytic leukemia cell exosomes deliver microRNA miR-146a to bone marrow MSCs, where miR-146a mediates the transition of MSCs to TAFs by targeting ubiquitin-specific peptidase 16 (USP16) [30].

### 2.2. Immunosuppression

MSCs are not able to activate T cell immune responses, since the levels of MHCI and MHCII antigens are reduced on the surface of MSCs. Additionally, there is also no expression of CD40, CD80, CD86 and co-stimulatory molecules necessary for T cell activation [31]; however, MSCs secrete a wide range of regulatory molecules, including IL-15, TGF-β1, and prostaglandin E2 (PGE2), which allows them to suppress the immune response by inhibiting dendritic cell (DC) maturation and suppressing the functions of T cells, B cells, and natural killer cells (NK cells), leading to a decrease in the secretion of soluble immune mediators and inhibition of NK-mediated cytotoxicity. The immunosuppressive properties of MSCs can also enhance tumor necrosis factor α (TNF-α), IL-1β, IL-6, and interferon (IFN) γ, which are involved in the development of antitumor immune responses and angiogenesis [32]. In some types of tumors, where the inflammatory microenvironment predominates, MSCs can directly interact with immune cells, which also leads to a weakening of antitumor immune responses [33]. For example, MSCs are able to inhibit T cell activity, either by suppressing their proliferation or, in the case of activated T cells, by inducing apoptosis. Inhibition of T cell proliferation can also be enhanced by various mechanisms, such as IFN-γ-mediated activation of the programmed cell death receptor ligand 1 (PD-L1) [34] or STRO-1 synthesis [35].

In silico analysis showed a positive association between MSC-associated genes and PD-L1 expression in various types of breast cancer. The conditioned medium (CM) of MSCs not only induced a phenotype switch, but also stimulated PD-L1 expression at the protein level through the secretion of various cytokines, especially CCL5. Treatment of MSCs with the cytokine inhibitor pirfenidone showed a significant decrease in CCL5 secretion and, hence, PD-L1 expression in breast cancer cells [36].

Presumably, the source of origin of MSCs may influence their immunosuppressive properties. For example, MSCs from adipose tissue and bone marrow inhibited lymphocytes but only when they were in close contact [37], while MSCs from Wharton’s jelly suppressed peripheral blood lymphocyte proliferation without the need for close contact [38].

Djouad et al. showed that the immunosuppressive effects of MSCs resulted in a higher incidence of melanoma in a mouse model with allogeneic melanoma cell transplantation [39]. Based on the known immunosuppressive properties of MSCs, it can be assumed that MSCs are able to suppress graft-versus-tumor and graft-versus-host reactions [40,41].

### 2.3. Angiogenesis Induction

A large number of pro-angiogenic factors, including VEGF, FGF2, PDGF, angiopoietins (ANGs), ephrins (EPHs), apelin (APLN) and their related receptors and chemokines, are known to promote tumor neovascularization [42]. These factors are often synthesized simultaneously, effectively interacting at different stages of tumor angiogenesis.

MSCs can stimulate tumor neoangiogenesis through the production and secretion of multiple angiogenesis factors such as ANGs, EGF, galectin-1 (GAL-1), IGF-1, keratinocyte growth factor (KGF), VEGF, TGF-β, IL-6, and macrophage inflammatory protein (MIP) 2. In addition, MSCs are directly involved in the recruitment of endothelial cells, thereby contributing to the formation of new tumor blood vessels [43,44]. Furthermore, studies have shown that MSCs can differentiate into endothelial cells, which also supports tumor vascularization [20].

In a xenograft model of mice using human hepatocellular carcinoma, it was found that human bone marrow MSCs were able to enhance tumor angiogenesis due to the action of TGF-β1, the secretion of which increased in tumor cells following the intravenous administration of MSCs. Modulation of the TGF-β1/SMAD signaling pathway and its interaction with VEGF may partly explain the complex role of MSCs in tumor progression [45].

Another study that demonstrated the effect of MSCs on angiogenesis was conducted by Huang et al. Using a xenograft model, with human colorectal cancer cells, MSCs and their cell mixture were injected subcutaneously into immunocompromised mice. Mixing various colorectal cancer cells with MSCs increased the tumor growth rate and angiogenesis to a greater extent than mixing with carcinoma-associated fibroblasts or normal colonic fibroblasts. The secretion of IL-6 from MSCs increased the secretion of endothelin-1 (ET-1) in cancer cells, which induced the activation of protein kinase B α (AKT) and ERK in endothelial cells, thereby increasing their ability to recruit and sustain angiogenesis in the tumor [46].

Batlle et al. found that inhibition of p38α kinase, a negative regulator of the MSC angiogenic program in the perivascular spaces, enhanced angiogenesis in human and mouse colon tumors and correlated with increased carcinogenesis [47]. It was also shown that p38α regulated the acquisition of endothelial-like phenotype of MSCs in colon tumors [47].

### 2.4. Epithelial–Mesenchymal Transition and Metastasis

Epithelial–mesenchymal transition (EMT) is the process by which epithelial cells change from an epithelial phenotype to a mesenchymal phenotype, and this occurs during embryonic development, wound healing, and pathological processes such as fibrosis and tumor progression [48]. Karnoub et al. found that human bone marrow MSCs, when co-administered subcutaneously with MCF-7 breast cancer cells, increased the metastatic potential of MCF-7 tumor cells in a xenograft mouse model with breast cancer [49]. MCF-7 cells stimulated de novo synthesis of CCL5 in MSCs, which then, using paracrine signaling, acted on tumor cells, enhancing their motility, invasion, and metastasis [49]. It was also shown that adipose tissue MSCs, when co-cultivated on Matrigel with MCF-7 cells, induced the formation of tumor spheres in vitro and promoted tumor growth in vivo in a xenograft mouse model with breast cancer. Tumor sphere formation by MCF-7 cells and MSCs has been associated with the induction of stem-like properties that mediate EMT [50].

The co-administration of adipose tissue MSCs and MDA-PCa-118b prostate cancer cells subcutaneously in mice increased tumor growth [51]. In addition, it was found that bone marrow MSCs stimulated the proliferation, migration and invasion of PC3 prostate cancer cells in vitro, and the described effect was then inhibited by blocking TGF-β [52]. In the early stages of carcinogenesis, TGF-β exhibited an immunosuppressive effect on tumor cells, by inhibiting cell proliferation, while at later stages it induced EMT, thus promoting tumor metastasis [53].

Lacerda et al. found that the co-administration of human bone marrow MSCs subcutaneously with SUM149 breast cancer cells increased the invasion and metastasis of the SUM149 cells in a xenograft model of mice with breast cancer [54]. Primary tumors injected together with MSCs were characterized by a higher level of EGF receptor synthesis and contributed towards the development of metastases following tumor removal. This effect was abolished by treatment with the EGF receptor inhibitor—erlotinib [54]. These results point to a role for MSCs in tumor progression and metastasis, possibly through the induction of EMT in primary tumor cells.

After subcutaneous co-injection with B16 mouse melanoma cells, MSCs were able to occupy perivascular sites in tumors and enhanced the metastasis of B16 cells to the lungs. MSCs activated an EMT-like profile in the B16 cells, increasing their mobility and invasiveness. These effects were abolished by blocking MET protein phosphorylation in B16 cells using small molecule inhibitors. MSCs also activated an EMT-like profile in human melanoma cells at various stages of progression. EMT activation in human cells has been associated with increased levels of phosphorylated (p) STAT1 and pSTAT3. Both murine and human melanoma cells are able to activate an EMT-like program and acquire metastatic features through activation of various pathways by MSC secretome [55].

Interestingly, in a study by Takigawa et al., the proliferation and migration of KM12SM human colon tumor cells increased following co-cultivation with MSCs. Expression of genes associated with EMT, such as FN, secreted protein acidic and rich in cysteine (SPARC), and GAL-1, were increased by direct co-cultivation with MSCs. Thus, MSCs induced EMT in colon cancer cells through direct intercellular contact and, therefore, may play an important role in colon cancer metastasis [56].

Human MSCs can promote hepatocellular carcinoma growth via activation of the mitogen-activated protein kinase (MAPK) signaling pathway, promoting metastasis in vivo via EMT. RNA sequencing showed an overexpression of α5 integrin (ITGA5) in the MSC-treated hepatocellular carcinoma cells. ITGA5 siRNA blocked the MSC-induced migration and invasion of hepatocellular carcinoma cells, whilst ITGA5 overexpression promoted tumor cell migration and invasion, indicating that ITGA5 expression is associated with MSC-induced tumor metastasis [57].

## 3. Antitumor Properties of Mesenchymal Stromal Cells

### 3.1. Apoptosis Induction

Sun et al. found that in a xenograft mouse model with breast cancer, systemic administration of MSCs from adipose tissue or umbilical cord blood inhibited metastasis and reduced the growth rate of MDA-MB-231 tumor cells [58]. The authors also showed that transplantation of MSCs at an early stage of tumor development, prior to detectable clinical symptoms, did not contribute to the growth and metastasis of tumor cells [58]. Therefore, tumor inhibition could be associated with the degradation of poly-(ADP-ribose) polymerase (PARP) due to the activation of caspase (CASP) 3, which induces apoptosis, and the expression of the *p21* gene [58,59].

Atsuta et al. found that when MSCs interacted with multiple myeloma (MM) cells, tumor cell proliferation was inhibited through Fas-mediated apoptosis (Fas and Fas-L are co-synthesized on MSCs, which, when co-cultivated, can reduce the proliferation of MM cells), and apoptosis was induced due to the activation of CASP3 and CASP8 [60]. Cord blood MSCs have inhibited the growth of H1299 lung cancer and A375 melanoma cells in vitro. At the same time, the synthesis of kinases, including AKT, phosphoinositide 3-kinase (PI3K), ERK, STAT3, and the mammalian target of rapamycin (mTOR), were significantly reduced in both tumor cell types when co-cultivated with MSCs [61,62]. Cord blood MSCs have also shown an inhibitory effect on ovarian cancer cells CAOV-3 in co-culture, where increases in the number of apoptotic CAOV-3 cells alongside decreased proliferation were noted [63].

The antitumor activity of particular MSCs may also depend on cell origin. For example, in a xenograft model of mice with human hepatocarcinoma, it was shown that when MSCs from bone marrow and umbilical cord blood were injected, the tumor size decreased, but MSCs from umbilical cord blood had a more pronounced antitumor effect than MSCs from bone marrow [64]. In addition, it was found that MSCs from various sources (adipose tissue, bone marrow, and umbilical cord blood) when co-cultivated with ovarian cancer cells (OVCAR3, CAOV-3, IGROV3, and SKOV3), caused a significant decrease in synthesis levels of tumor markers, such as tumor antigen CA-125, lactate dehydrogenase (LDH) and β-subunit of human chorionic gonadotropin (hCG) in tumor cells, and also suppressed cell proliferation in vitro. A similar effect was observed when ovarian cancer cells were cultivated with CM from MSCs [65]. It was found that the CM from adipose tissue MSCs suppressed MCF-7 breast cancer cell growth by activating the STAT1 signaling pathway with IFN-β [66].

Coccè et al. showed that MSCs, their lysate, and secretions, inhibited proliferation of malignant pleural mesothelioma cells. MSC lysates also induced apoptosis of tumor cells. In addition, MSCs integrated with tumor cells and had a significant inhibitory effect on the proliferation of malignant pleural mesothelioma cells [67]. Aslam et al. showed that MSC lysates (Wharton’s Jelly and bone marrow) induced a general inhibitory effect on the proliferation of glioma cells and fibroblasts [68].

MSCs obtained from the placental decidual parietal membrane modulated the expression of various proapoptotic genes, as well as oncogenes, in MDA231 human breast cancer cells. The MDA231 cells showed a significant reduction in the proliferation, migration and invasion potential after they were treated with MSCs [69].

Thus, a large number of studies confirm that MSCs can induce apoptosis through various mechanisms; however, the use of MSCs in therapy requires further study of their effect on tumor cells in order to identify specific mechanisms and select certain conditions for their interaction with tumor cells.

### 3.2. Regulation of Cell Signaling

PI3K/AKT and WNT/β-catenin signaling pathways control cell survival, proliferation, growth, migration, and metabolism. Numerous studies have described the need for AKT signaling for tumor cell migration, invasion, and survival. The WNT signaling pathway is also associated with the development of carcinomas of the breast, liver, colon, skin, stomach, and ovaries [70].

Intravenous administration of MSCs effectively inhibited tumor cell proliferation via inhibition of the AKT pathway in a model of Kaposi’s sarcoma [71]. Moreover, cord blood stem cells activated the synthesis of phosphatase PTEN (phosphatase and tensin homolog deleted on chromosome 10) in glioma cells, which led to the suppression of signal transduction along the AKT pathway [72].

In addition to inhibiting the PI3K/AKT signaling pathway, MSCs can also suppress the WNT/β-catenin signaling pathway by inducing Dickkopf-related protein 1 (DKK-1) synthesis. In vitro experiments showed β-catenin levels decreased in human carcinoma cells (hepatocellular carcinomas H7402 and HepG2, breast carcinomas MCF-7, hematopoietic carcinomas K562 and HL60) under the influence of DKK-1 secreted by MSCs. Suppression of DKK-1 activity with neutralizing antibodies or small interfering RNAs (siRNAs) resulted in a weakening of the inhibitory effect of MSCs on tumor cell proliferation [73,74,75]. The study’s authors showed that MSCs can also affect the STAT3 pathway in breast cancer cells. CM from human umbilical cord MSCs suppressed the activation of STAT3 signaling and tumor growth [76].

Thus, the regulation of signaling pathways by MSCs can lead to tumor inhibition and the study of these properties of MSCs can provide new clues to the MSC-mediated mechanism of disease prevention.

### 3.3. Cell Cycle Regulation

MSCs secrete many cytokines that can temporarily induce cell cycle arrest in tumor cells in the G0/G1 phase by reducing the synthesis of cyclins A, E, and D2, as well as p27KIP1 [77,78]. Lu et al. found that adipose tissue MSCs and CM from these cells suppressed the growth of pancreatic adenocarcinoma [59]. In addition, CM from MSCs stimulated tumor cell necrosis after G0/G1 phase arrest in the absence of apoptosis, and MSCs modulated the expression of *p21* gene, which is a negative regulator of the tumor cell cycle [59].

In another experiment, the co-cultivation of chronic myelogenous leukemia K562 cells with bone marrow or umbilical cord blood MSCs resulted in the accumulation of tumor cells in the G0/G1 phase and a slowdown in the transition to the S phase [79,80]. This regulation of the cell cycle may be caused by the secretion of cytokines such as IL-6 and IL-8 [80].

Sarmadi et al. showed that human umbilical cord MSCs affected the proliferation of leukemic cells—the growth of tumor cells was stopped at the G0/G1 phase. This result may be associated with the impaired expression of genes encoding cell cycle regulators, such as cell division protein kinase 6 (CDK6), cyclin E2 (CCNE2), cyclin-dependent kinase inhibitor (CDKN) 1A, CDKN2A, dumbbell former 4 protein (DBF4), mouse double minute 2 homolog (MDM2), proteasome activator complex subunit 3 (PSME3), and G0/G1 switch 2 (G0S2) [81].

In contrast, many in vivo studies have shown that the combined administration of MSCs and tumor cells resulted in more active tumor growth than when only tumor cells were administered; however, the exact mechanisms by which the MSCs are able to induce cell cycle arrest are not fully understood. Possibly, the slowing down or stopping of the cell cycle can be induced in certain types of tumor cells and under certain conditions of co-cultivation (type of medium, cell concentration or time of co-cultivation).

### 3.4. Effect on the Immune Response

It is known that MSCs can suppress immune responses; however, there are data reporting the opposite effects of MSCs too. For example, it has been shown that MSCs can stimulate peripheral blood mononuclear cells [82,83]. Kawabata et al. showed that rat umbilical cord MSCs attenuated mammary tumor growth by enhancing the host’s antitumor immune response. Immunohistochemical analysis showed that the majority of infiltrating lymphocytes in tumors from MSC-treated rats were CD3+ T cells. In addition, the MSC treatment significantly increased the infiltration of CD8+ and CD4+ T cells and NK cells throughout the tumor tissue [84] and it should be noted that the presence of an immune infiltrate is usually associated with a good prognosis. Ohlsson et al. found that the co-administration of tumor cells and MSCs to rats induced increased infiltration of granulocytes and monocytes in vivo, as opposed to the administration of either tumor cells or MSCs alone [85]. The authors used a preformed gelatin matrix containing colon carcinoma cells and MSCs, which was then subcutaneously transplanted into rats to control tumor growth and a subsequent inflammatory response. MSCs suppressed the proliferation of tumor cells and infiltration by both granulocytes and macrophages was much higher in rats that were simultaneously transplanted with a mixture of tumor cells and MSCs than in rats injected with tumor cells without MSCs [85].

According to the literature, it is possible to influence MSCs by blocking their immunosuppressive properties. For example, inhibition of indoleamine-pyrrole 2,3-dioxygenase (IDO), heme oxygenase (HO), arginase (ARG) I and ARGII, nitric oxide synthase 2 (NOS2), PGE2 and the TGF-β, PD-1-PD signaling pathway -L1 may lead to overcoming immunosuppression in TME [86,87,88,89,90].

Thus, the effect on the immune system and its stimulation can be used as one of the options for antitumor therapy; however, a large amount of data on the immunosuppressive properties of MSCs has cast doubt on the possibility of their use for such therapy. Therefore, it is relevant to understand the mechanisms of the influences of MSCs on the immune system, since the knowledge gained can accelerate the development of this direction.

## 4. Vesicles of Mesenchymal Stromal Cells: Structural Features Mediated by Origin

Extracellular vesicles (EVs) are a heterogeneous group of membrane structures that form almost all cells of the body and are involved in intercellular communication [91]. EVs include exosomes, microvesicles (MVs), and apoptotic bodies (ABs) [92] (Table 1). ABs formed during programmed cell death are used to package the contents of the cell and often include elements of nuclear material and organelles. The formation of ABs is an important process that confirms cell death by apoptosis. ABs contain various cellular components, namely, micronuclei, chromatin remnants, parts of the cytosol, degraded proteins, DNA fragments, and a large amount of RNA. This content may have an effect on the surrounding cells; however, the effect of ABs on the cellular microenvironment is yet to be studied [93]. In this review, ABs will no longer be discussed, and the focus will be on exosomes and MVs.

Exosomes are formed inside the cell through the endosome. If an endosome is labeled with lysobisphosphatidylic acid (phosphatidylinositol-3-phosphate) and contains ubiquitinated proteins, it will be degraded by lysosomes. The presence of ceramides on the endosome membrane is a signal for exosome exit from the cell [94]. The production of MVs occurs due to the disorganization of the cytoskeleton and internal changes in the cell. The release of MVs is induced by increases in intracellular Ca^2+^ levels, which are mediated by phospholipid distribution changes in the plasma membrane. This increase promotes the transfer of phosphatidylserine from the inner part of the membrane to the outer cell surface [95], moreover, this activity is usually associated with the recruitment and activation of the Ca^2+^-dependent scramblase enzyme [96] and modification of the cytoskeleton [97] through ATP-dependent mechanisms [98].

The composition of EVs depends on the parental cell type [99]. EVs contain cytoplasmic proteins, cytoskeletal proteins, and surface receptors [100]. Their membranes are enriched with cholesterol, sphingomyelin, glycosphingolipids and phosphatidylserine, which maintain the stability of these structures, protecting the EV contents from degradation by extracellular enzymes and promoting their circulation in the human body within blood and lymph flow [101]. EVs are key mediators between tumor cells and TMEs due to their ability to readily transport various molecules, including lipids, transcription factors, mRNAs, non-coding regulatory RNAs, and proteins [102].

Notably, EVs are capable of receptor interactions with the body’s cells. The mechanism of the fusion and transfer of molecules can be carried out through clathrin-mediated and caveolin-dependent endocytosis, due to ligand/receptor interactions. This interaction seems to be a cell-specific mechanism and can lead to the activation of various biological processes, such as signal transduction, endocytosis, and membrane fusion, whereas macropinocytosis and phagocytosis are less common [103]. The fusion of EVs and cells may depend on the lipid composition and on the acidity of the environment [104] and because EVs can transmit “molecular information”, they are involved in a variety of bodily processes, including inflammation, blood clotting, and carcinogenesis [105].

**Table 1:** Characterization of extracellular vesicles.

Type of EVs	Size	Proteins Involved in Biogenesis of EVs	Functions	Isolation Methods
Exosomes	40–150 nm [106]	Rab proteins, endosomal sorting complexes required for transport (ESCRT), syndecan (SDC), syntenin (SDCBP), autophagy related 12 (ATG12), tetraspanins (TSPANs), tumor susceptibility gene 101 (TSG101), Alix, neutral sphingomyelinase 2 (nSMase2), synaptotagmin-7 (SYT7) [107,108]	Produce intercellular communication and influence the microenvironment [109]	- Differential centrifugation including ultracentrifugation (at 100,000× *g*) - Purification by density gradient using sucrose gradients - Filtration - Immune-isolation - Size-exclusion chromatography - Fluorescence-activated cell sorting (FACS) - Use of cytochalasin B [110,111,112,113,114,115,116,117]
Microvesicles	150–1000 nm [106]	Acid sphingomyelinase (ASMase), transmembrane protein 16F (TMEM16F), ADP-ribosylation factor 6 (ARF6), arrestin domain containing 1 (ARRDC1), floppases and flippases, phospholipase D (PLD), ERK, myosin light-chain kinase (MLCK) [107,108]		
Apoptotic bodies	50–2000 nm [106]	Annexin V, CASP3, thrombospondin (TSP), C3b [107,110]	Play a role in the destruction of apoptotic cells, and presumably can participate in the transfer of information between cells [109]	

MSC extracellular vesicles (EV-MSCs), such as exosomes, have membranes enriched in lipid raft elements such as GM1 gangliosides and transferrin receptors. They are rich in annexins, TSPANs (CD63, CD81, and CD9), lactadherin, and heat shock proteins (Hsp60, Hsp70, and Hsp90), contain clathrin, calveolins, and endosome-specific proteins such as Alix and Tsg101, cell-type specific proteins [118] and lysosome-associated membrane glycoprotein 2 (Lamp2B) [119]. Exosomes carry characteristic lipids and contain cholesterol, ceramide, diacylglycerol, sphingomyelin, and phosphatidylserine. MSC microvesicles (MV-MSCs) lack endocytic pathway proteins, but are high in phosphatidylserine, contain a protein associated with lipid rafts such as integrins and flotilins, and are enriched in cholesterol, sphingomyelin, and ceramide [120]. In addition, MSC vesicles carry membrane proteins, including surface markers that are used to characterize MSCs such as CD13, CD29, CD44, CD73, CD90, and CD105. [17,121].

Interestingly, several molecules that make up the lipid portion of EV-MSCs, such as diacylglycerol (DAG), sphingomyelin (SM), and ceramides, are involved in signaling pathways and energy homeostasis [122]. Ceramide is a known tumor suppressor that blocks the cell cycle and induces apoptosis. In addition, tumor cells are able to activate enzymes that redirect ceramide metabolism towards mitogenicity [123]. SM is a key component of lipid rafts, which are important signaling platforms [124], and changes in SM levels within the cell membrane can modify signaling pathways. Decreases in SM levels have been associated with the aggressive transformation of tumor cells mediated by the activation of oncoproteins and modifications of the membrane composition [125]. It should be emphasized that DAG mediates key signaling pathways by inducing several classes of proteins, such as protein kinase C (PKC), which regulate various processes associated with proliferation, apoptosis, migration, and tumorigenesis [126]. These data clearly demonstrate that bioactive lipids carried by EV-MSCs into target cells can modulate various cellular processes involved in tumor physiology [17].

As mentioned earlier, EVs can carry a variety of molecules. It has been shown that the molecules present in EV-MSCs can be divided into several groups depending on their molecular and cellular function. These include transcription factors, extracellular matrix (ECM) proteins, chemokines, cytokines, enzymes, growth factors, RNA-binding molecules, miRNAs, and molecules involved in angiogenesis, cell adhesion, development, degradation, protein folding, immunomodulation, regulation of apoptosis, and other functions [127]. For example, the CC-motif chemokine receptor-2 (CCR2) has been shown to be expressed on exosomes derived from Strain Balb/c mice bone marrow MSCs. Exosomes with high expression levels of CCR2 can reduce the concentration of free CCL2 and suppress its function relating to recruiting or activating macrophages [128]. Expression of IL-5, IL-6, IL-10, IL-13, and serpin E1 (also known as plasminogen activator inhibitor-1, PAI-1) have also been detected in EV-MSCs [116]. In addition, MMP-2, which exhibited functional enzymatic activity and was transferred to MCF-7 breast cancer cells [129], WNT4, which activates the WNT/β-catenin pathway and triggers angiogenesis [130], PDGF receptor β (PDGFR-β), tissue inhibitor of metalloproteinase (TIMP) 1 and 2, and lactic and glutamic acids were also identified [122].

It has been shown that the transfer of miR-146a by exosomes from MSCs pretreated with IL-1β can affect immunomodulatory properties. Exosomal miR-146a was translocated into macrophages, resulting in M2 polarization and increased survival in septic mice. Inhibition of miR-146a leads to the absence of immunomodulatory properties of exosomes [131]. Baglio et al. compared small RNA expression profiles dominated by miRNAs and snoRNAs between exosomes released by adipose tissue and bone marrow MSCs and miR-21-5p, miR-22-3p, miR-10b-5p, and miR-222-3p were among the most abundant in both cells and exosomes. Differences were found in the sorting of evolutionarily conserved tRNA species, which were apparently associated with the tissue origin and stemness of MSCs [132]. Gong et al. studied the proangiogenic properties of exosomes using a tube-like structure formation and spheroid-based sprouting of human umbilical vein endothelial cells (HUVECs). The addition of exosomes contributed to tubular structure formation by HUVEC in vitro and increased blood flow inside Matrigel implanted in mice due to the transfer of pro-angiomiRs. Suppression of miR-30b reduced the pro-angiogenic capacity of exosomes [133].

Proteomic studies of EVs are important. The protein content of MSCs and their exosomes were characterized using mass spectrometry with conjugated liquid chromatography. In total, 6342 proteins were identified in MSCs and 1927 in exosomes [134]. In addition, the MV-MSC proteome showed molecules that could be presumably related to their potential to repair damaged tissues [135].

All the molecules contained within EVs could affect tumor cells in different ways. There are studies that describe the dual effects of EV-MSCs on tumor growth in both in vitro and in vivo models. Bailey et al. conducted a systematic review and meta-analysis of the available data to clarify some trends in the effects of EVs on tumor growth. All of the preclinical controlled studies investigating the effects of EV-MSCs on tumor growth were identified for this review. The potential risk of bias was assessed using the SYRCLE instrument. A meta-analysis of the random effects of reduction in tumor mass/volume was performed, and 29 articles and 22 reported tumor response datasets were identified and included in the meta-analysis. EV-MSCs have shown a mixed response to tumor progression, with some studies reporting tumor growth inhibition and others reporting tumor progression. EV-MSCs that overexpressed antitumor RNAs were associated with significant tumor reduction in a meta-analysis; however, the existing differences in the results may be related to the design of the experiment, the MSC tissue origin, EV isolation method, their individual characteristics, dosing and administration scheme, and even the tumor cell type [136].

Therefore, a large number of studies have indicated the possibilities relating to transporting various molecules via EV-MSCs and have proved they have an influence on the microenvironment.

## 5. Pro-Tumor Properties of Mesenchymal Stromal Cell Derived Extracellular Vesicles

EV-MSCs contain and carry proteins associated with tumor growth and ECM remodeling (TIMP-1, TIMP2, MMP-9, MMP-2), angiogenesis (VEGF and PDGF) [122], proliferation (EGF, TGF-β, FGFs), cytokines including IL-6, IL-8 and IL-1β [137], RNA and microRNA (Table 2). Exosomes derived from MSCs carrying growth factors and IL-6 also promote the proliferation of multiple myeloma cells in vitro and in vivo [138].

It was shown that vesicular miR-21 stimulates renal cell carcinoma and breast cancer proliferation [122]. Human umbilical cord EV-MSCs promoted the growth of LUAD cells, which the authors attribute to the exosomal delivery of miR-410 to LUAD cells and a decrease in PTEN expression [139]. EV-MSCs have also significantly reduced chemotherapy-induced apoptosis. For example, microRNA sequencing was used to identify hsa-miR-11401, which affects p53-dependent cell apoptosis [140].

Ren et al. studied the mechanism contributing to the development of lung cancer. In this study, vesicles were isolated from human bone marrow MSCs under normoxia and hypoxia culture conditions. Overexpression of PTEN, PD-4, and reversion-inducing cysteine-rich protein with Kazal motifs (RECK) in A549 cells significantly reduced the anti-apoptotic and prometastatic effects. EV injection significantly increased tumor growth, tumor cell proliferation, intratumoral angiogenesis, and polarization of M2 macrophages in vivo partially via miR-21-5p [141]. The differentiation of monocytic myeloid suppressor cells into highly immunosuppressive ones was induced by MSC exosomes affected by the tumor. They have been shown to contain TGF-β, C1q, and semaphorins, which promote myeloid tolerogenic activity by driving PD-L1 overexpression. These exosomes contribute to the accelerated progression of breast cancer [142].

Incubation of MCF-7 breast cancer cells with EV-MSCs resulted in proliferation, reduced migration, and improved cell adhesion, both in a monolayer culture of these cells and in a 3D model. This study confirmed the ability of EV-MSCs to maintain a dormant state of tumor cells [143].

The effect of EV-MSCs on tumor cells can be triggered through the activation of various signaling pathways. For example, it has been shown that the Wnt signaling pathway is activated in MCF-7 cells after the addition of MSC exosomes [144]. MSC exosomes triggered VEGF expression in tumor cells by activating the ERK1/2 pathway. The inhibition of ERK1/2 activation or RNase pretreatment of exosomes abolished these effects, suggesting a critical involvement of exosome-delivered mRNAs in stimulating angiogenesis [145]. Exosomes derived from human bone marrow promoted the growth of MG63 and SGC7901 cells through activation of the Hedgehog signaling pathway. Inhibition of the Hedgehog signaling pathway significantly suppressed the tumor growth process [146]. Additionally, exosomes from human umbilical cord MSCs treated with human breast cancer cells (MDA-MB-231 and MCF-7) promoted EMT via the ERK pathway. This led to the increased proliferation, migration and invasion of tumor cells [147]. In addition, MSC exosomes have been shown to enhance the migration and invasion of HGC-27 cells by inducing EMT and activating the AKT pathway [148].

Mao et al. found that the state of p53 in MSCs can influence tumor progression. It has been shown that ubiquitin protein ligase E3 component n-recognin 2 (UBR2) is highly expressed in p53−/− MSCs of mouse bone marrow and their exosomes. These exosomes induce UBR2 overexpression in p53+/+ MSCs from mouse bone marrow and mouse carcinoma (MFC). The WNT/β-catenin pathway is activated, using these exosomes, leading to cell proliferation, migration and the expression of genes associated with stemness [149].

Thus, these studies demonstrate that the transfer and uptake of EV-MSCs by tumor cells can trigger the mechanisms involved in the progression of cancer.

**Table 2:** Summary of current data on the effect of EVs on tumor progression.

MSC Source	Type of Tumor	Reference
Bone marrow	Multiple myeloma	[138]
Bone marrow	Renal carcinoma	[122]
Umbilical cord	Lung adenocarcinoma	[139]
Bone marrow	Lung adenocarcinoma	[141]
Adipose tissue	Breast adenocarcinoma	[144]
Bone marrow	Osteosarcoma and gastric carcinoma	[146]
Umbilical cord	Breast adenocarcinoma	[147]
Umbilical cord	Gastric carcinoma	[148]

## 6. Antitumor Properties of Mesenchymal Stromal Cell Derived Extracellular Vesicles

EV-MSCs are carriers of cell content, including certain substances associated with the induction of antitumor activity in cells [150,151]. Evidence relating to EV antitumor activity has already been obtained.

Bruno et al. discovered that MVs from human bone marrow MSCs inhibited cell cycle development and induced apoptosis in HepG2 and Kaposi cells and necrosis in Skov-3 cells. Microarray assays showed that MVs triggered the expression of genes involved in the development of the cell cycle, while proteins such as GTP-binding RAS-like 3 (DIRAS3), retinoblastoma-like 1 (RBL-1), and cyclin-dependent kinase inhibitor 2B transcript (CDKN2B), which are related to the anti-proliferative pathway, were upregulated. Moreover, RBL-2 is a negative cell cycle regulator that increased following MV exposure. The gene array profile of cells treated with MVs also showed upregulation of the following transcripts: baculovirus IAP repeat containing 5 (BIRC5), DEAD/H-box helicase 11 (DDX11), and component 5 of the minichromosome maintenance complex (MCM5). To confirm the effect of MVs on molecular changes, cells were treated with a transcription inhibitor (ActD). As a result, it was proved that for the most part the identified transcripts were due to de novo gene expression induced by the inclusion of MVs. In HepG2 cells, some of the tested transcripts (RBL-1, CDKN2B and DIRAS3) showed a significant difference between ActD-treated cells in the presence or absence of MVs, which also confirms the functional transfer of MVs transcripts [152].

The ability of MV-MSCs from human Wharton’s jelly to inhibit the growth of bladder tumor T24 cells by inhibiting AKT phosphorylation and activating cleaved CASP3, p-p53 and p21 was evaluated. The amount of Ki-67 protein, which is used as a marker of cell proliferation, was also investigated. In T24 cells treated with MVs, a decrease in the amount of this protein was found compared to control cells, which could indicate that the cells were in a dormant state (G0/G1a phase of the cell cycle) [153].

Kalimuthu et al. evaluated the antitumor effects of mouse bone marrow EV-MSCs on Lewis lung carcinoma (LLC). Apoptosis induction has been shown in LLC-effluc cells and 4T1. Decreased pERK and increased cleaved CASP3 and cleaved PARP have been shown [154].

In all of these studies, the effect of EVs were confirmed in in vivo models which exhibited tumor growth inhibition when EV-MSCs were added [152,154]. Notably, Kalimuthu et al. developed a method for improved visualization of EVs in vivo, involving the use of Renilla luciferase (Rluc). Bioluminescent EVs were obtained by transduction of mouse bone marrow-derived MSCs with lentiviral vectors containing the Rluc gene and isolation of EVs from transduced cell culture supernatants. The method provided more accurate data on the distribution of vesicles in vivo [154].

The antitumor role of EV-MSCs also lies in the inhibition of tumors via activation of immune cell functions [155]. MVs exhibit the ability to inhibit autoreactive lymphocyte proliferation and stimulate their secretion of anti-inflammatory cytokines IL-10 and TGF-β, and to additionally stimulate the generation of CD4+CD25+Foxp3+ regulatory T cells [156]. Ko et al. showed that exosomes obtained from the culture medium of adipose tissue MSCs can contribute to the antitumor immunity of NKT cells. In this study, a model of hepatocellular carcinoma in rats was used. Compared to controls, the exosome-treated rats had a significantly higher percentage of circulating NKT cells at days 5 and 15 post-treatment. The exosome-treated rats also showed significant tumor reduction compared to the controls [157].

The antitumor effects exerted by exosomes may be due to the transfer of various miRNAs (Table 3). For example, Lee et al. observed that vesicles from mouse bone marrow MSCs suppressed angiogenesis by suppressing VEGF expression in tumor cells due to the presence of miR-16 in exosomes [158]. Reza et al. showed that exosomes derived from human adipose tissue MSCs are important miRNA carriers and were able to induce apoptotic signaling via the activation of various pro-apoptotic signaling molecules, such as B-cell lymphoma 2 (BCL2) associated X (BAX), CASP9 and CASP3, as well as suppression of the anti-apoptotic protein BCL2 [159].

The work conducted to date on EV-MSC content and understanding the molecular mechanism of the MSC action and their EVs on the tumor and its microenvironment, helps provides data for the development of new antitumor therapeutic strategies.

Seyhoun et al. were able to demonstrate that co-treatment with sorafenib alongside MSC CM was able to enhance the cytotoxic effects of sorafenib on HepG2 cell lines [169]. The combination of sorafenib with MSCs showed a similar result, which included the inhibition of hepatocellular carcinoma growth in a mouse model [170]. In addition, MSC secretome from adipose tissue has been shown to play a role in reducing the cell viability of triple-negative breast cancer in a dose-dependent manner [171].

Interestingly, EVs can be modified in a variety of ways. For example, Qui et al. proved that the incorporation and release of cabazitaxel from MSC exosomes inhibited PI3K, AKT, and mTOR activation in oral squamous cell carcinoma. The antitumor effect of exosomes with cabazitaxel was also confirmed in vivo [172]. Intravenous injections of MSC exosomes loaded with taxol have demonstrated their ability to reduce breast tumor in vivo. Similarly, epirubicin exosomes exhibited cytotoxic effects in various tumor cell cultures [173], while Wei et al. used exosomes isolated from MSCs as drug nanocarriers for doxorubicin (DOX) loading. Exosomes with DOX were taken up by MG63 osteosarcoma cells and caused their death [174]. Exosomes with meta(tetrahydroxyphenyl)chlorin caused tumor necrosis and promoted the infiltration of antitumor immune cells. These vesicles contributed to an increased lifespan in a mouse model of colorectal carcinomatosis [175].

EVs can be obtained from genetically modified MSCs. For example, induced membrane vesicles from MSCs that overexpress TNF-related apoptosis-inducing ligand (TRAIL), PTEN, and IFN-β1 were able to activate human immune cells and induce apoptosis in various types of carcinomas in vitro [117]. Induced membrane vesicles derived from genetically modified MSCs with IL-2 have contributed towards the activation of human CD8 T-killers and the suppression of human triple negative cancer MDA-MB-231 [112].

Multiple doses of exosomes derived from the supernatant of engineered MSCs with TRAIL (Exo-TRAIL) reduced melanoma size in mice [176]. You et al. used EVs from MSCs transfected with an adenoviral vector with a gene encoding lipocalin type prostaglandin D2 synthase (L-PGDS). EVs-L-PGDS inhibited gastric cancer growth, induced cell apoptosis, reduced the expression of stem cell markers including Oct4, Nanog, and Sox2, and inhibited STAT3 phosphorylation in SGC-7901 gastric cancer cells [177].

An exosomal platform was created that includes synthetic polyvalent antibodies (SMART-Exo) under work conducted by X. Shi et al. This platform functions as an artificial cellular immunity modulator to redirect immune effector cells and control their immunoreactivity. SMART-Exos target CD3 receptors and human epidermal growth factor receptor 2 (HER2). They not only recruit human T cells to HER2-positive breast cancer cells, but also cause highly efficient and specific death of HER2-expressing breast cancer cells. Importantly, in vivo studies using mouse xenograft models support the antitumor activity of SMART-Exos. This study presents a SMART-Exos-based targeted immunotherapy strategy for HER2-positive breast cancer and demonstrates SMART-Exos as a broadly applicable platform for the development of cell-free oncology therapies [178].

It is possible to target EVs to a specific tumor type. For example, Wang et al. used cholesterol to conjugate the AS1411 aptamer to EVs to confer tumor targeting capability. The ability of AS1411 aptamer-modified EVs to deliver let-7 siRNA or VEGF siRNA to nucleolin-expressing cancer tissues was evaluated. High antitumor activity was found for AS1411-EV, loaded with the lethal-7 gene microRNA precursor, let-7, or VEGF siRNA in vitro and in vivo [179].

The evidence from these studies indicates that the use of EVs in antitumor therapy has prospects. EV-MSCs can be transformed and used as therapeutic agents, and EV-MSCs can also be used as a delivery vehicle for deficient substances for cells or drugs. EVs transport the substances loaded into them due to the membrane structure; however, many aspects of their application still remain unexplored.

## 7. Different Effects of Mesenchymal Stromal Cell Derived Extracellular Vesicles

There are studies highlighting that EV-MSCs have different effects on tumor cells. For example, exosomes were isolated from the CM of bone marrow MSCs obtained either from patients with MM or from healthy donors. Exosomes of MSCs from MM patients had higher levels of oncogenic proteins, cytokines, and adhesion molecules compared to exosomes of MSCs from healthy donors. These exosomes from two sources were cultured with MM cells. As a result, exosomes of MSCs from MM patients promoted the growth of MM tumors, and exosomes of MSCs from healthy donors inhibited the growth of MM cells [138]. These data are consistent with the study by Dabbah et al. where the role of MV-MSCs from bone marrow of healthy donors and patients with MM was studied on MM cell cultures. Within 24 h, MV-MSCs were internalized within the MM cells, eliciting opposite responses according to the origins of the MVs. MV-MSCs from healthy donors decreased viability, proliferation, migration, and TI (translation initiation, including an increase or decrease in phosphorylated factors peIF4E and peIF4GI) (↓ 15–80%; *p* < 0.05), while MV-MSCs from patients with MM increased them (↑ 10–250%, *p* < 0.05) [180].

Del Fattore et al. compared the effects of exosomes from three sources, including bone marrow (BM), umbilical cord (UC), and adipose tissue (AT), on the U87MG glioblastoma cell line. BM- and UC-EV-MSCs reduced cell proliferation, while the opposite effect was observed with AT-EV-MSCs. Moreover, both BM- and UC-EV-MSCs induced apoptosis of glioblastoma cells, but no such effect was found in AT-EV-MSCs [181]. Du et al. showed that MVs from human Wharton’s jelly MSCs inhibit the growth of bladder cancer cells; however, these MVs promoted the growth and aggressiveness of RCC (786-0) kidney cancer cells in vitro and in vivo [182]. Vallabhaneni et al. investigated the role of human EV-MSCs in breast cancer metastasis using the MDA-MB-231 parental cell line and organotropic sub-lines (231BrM-2a, 231LM-4175, 231BM-1833). It was shown that EV-MSCs significantly suppressed the metastatic potential of the parental cell line compared to their organotropic sublines [183].

Thus, the question of the effect of EV-MSCs on tumor cells still remains open. Even EV-MSCs from the same origin can affect tumor cells of different types in opposite ways. The use of EV-MSCs as a therapeutic agent is undoubtedly of interest; however, such a dual role of EV-MSCs hinders their application. Perhaps, the selection and EV-MSC use to create a therapeutic drug need to be carried out individually for each tumor type.

## 8. Conclusions

At present, the involvement of MSCs in carcinogenesis and their functions in TME are actively being studied. The ability of MSCs to influence the tumor process, depending on various factors, is of interest for the use of these cells in antitumor therapies; however, the mechanisms behind the suppressor and stimulatory effects of MSCs are not yet fully understood. Despite the available publications proving the antitumor properties of MSCs, most studies indicate that MSCs can be recruited into the TME, promoting its growth and metastasis, as well as immunosuppression.

One of the ways to overcome the undesirable effects of MSC-based therapy can be the use of EVs. The molecular mechanisms behind the effects of EV-MSCs on tumor cells have not yet been sufficiently investigated. Opposite responses of tumor cells to the addition of EV-MSCs may depend on the triggering of different signaling pathways. These signaling pathways can be activated or inhibited by substances that are carried by EV-MSCs. Therefore, a detailed study of the molecules carried by EV-MSCs seems to be relevant. In addition, the differing responses by tumor cells may be associated not only with the origin of MSCs and the type of cancer, but also with the design of the study, the dose of EVs, the method of administration, and the protocol used for obtaining the EVs. EV isolation methods such as differential centrifugation, sucrose gradients, microfiltration, antibody-coated magnetic beads, and microfluidic devices can isolate subpopulations of EVs that are differently enriched in certain groups of proteins, mRNAs, and miRNAs that affect tumor cells in opposite ways. It is possible that the choice of a specific protocol for the EV isolation and adherence to certain established general rules for their characterization and use for studies on tumor cells will allow for the creation of an improved antitumor therapy and a new drug for the treatment of oncological diseases in the future.

## Figures and Tables

**Table 3:** MicroRNAs transported by vesicles and therapeutic effect.

miRNAs Included in EV-MSCs	Effect on Tumor
miR-16	Suppression of VEGF in human nasopharyngeal carcinoma cells (anti-angiogenic activity) [20,158,160].
miR-100	Suppression of VEGF production and thus angiogenesis in breast cancer cells through modulation of mTOR/hypoxia-inducible factor 1α (HIF-1α) [161].
miR-146b	Binds to EGFR mRNA and ultimately reduces the growth, migration and invasion of cancer cells in culture [162].
miR-143	Reduction in osteosarcoma cell migration [163].
miR-9-3p	Inhibition of the endothelial cell specific molecule 1 (ESM1) tumor promoter gene in bladder cancer [164].
miR-379	Reduction in the rate of tumor formation and growth in vivo. It is a potent tumor suppressor in breast cancer, mediated by the regulation of SRY (sex region Y)-box 2 (SOX-2) [165].
miR-340	Inhibition of angiogenesis via the hepatocyte growth factor/c-MET (HGF/c-MET) signaling pathway in endothelial cells [166].
miR-199a-3p	Inhibition of tumor growth and HepG2 cell migration by targeting CD151, integrin α3 and 6 [167].
miR-16-5p	Decreased proliferation and migration of tumor cells and increased apoptosis of tumor cells [168].

## Data Availability

Not applicable.
